# Supplementary material for: Functional Analysis of Alkaline Phosphatase in Whitefly Bemisia tabaci (Middle East Asia Minor 1 and Mediterranean) on Different Host Plants
Source: Genes (Basel). 2021 Mar 29;12(4):497. doi: 10.3390/genes12040497 (PMC8065556; doi:10.3390/genes12040497)
Supplement: Supplementary file 1 [file genes-12-00497-s001.zip › Supplementary figures_HWH_24032021.docx]

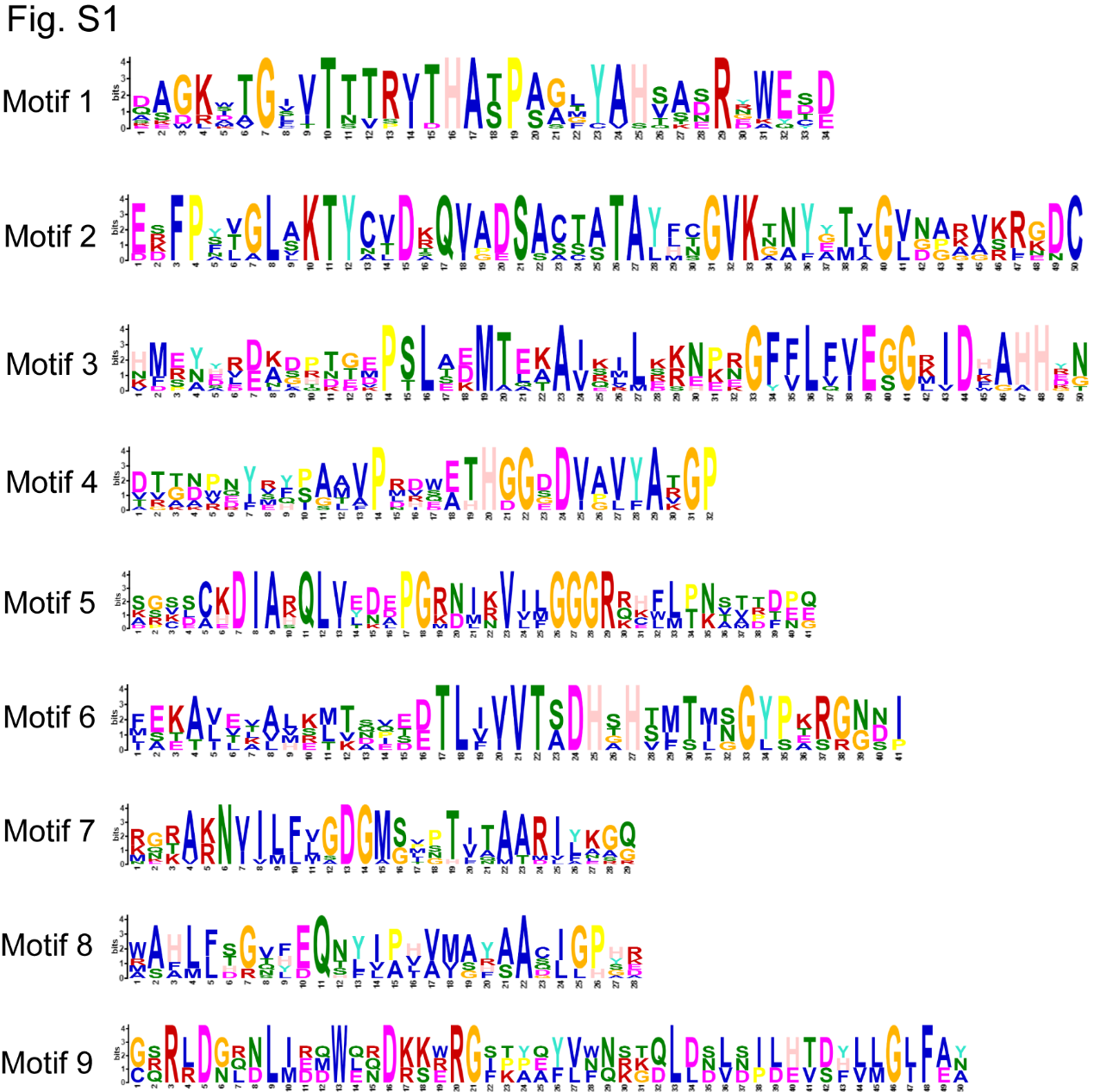


Fig.S.1 All motifs were identified by the MEME database with the complete amino acid sequences of ALPs.


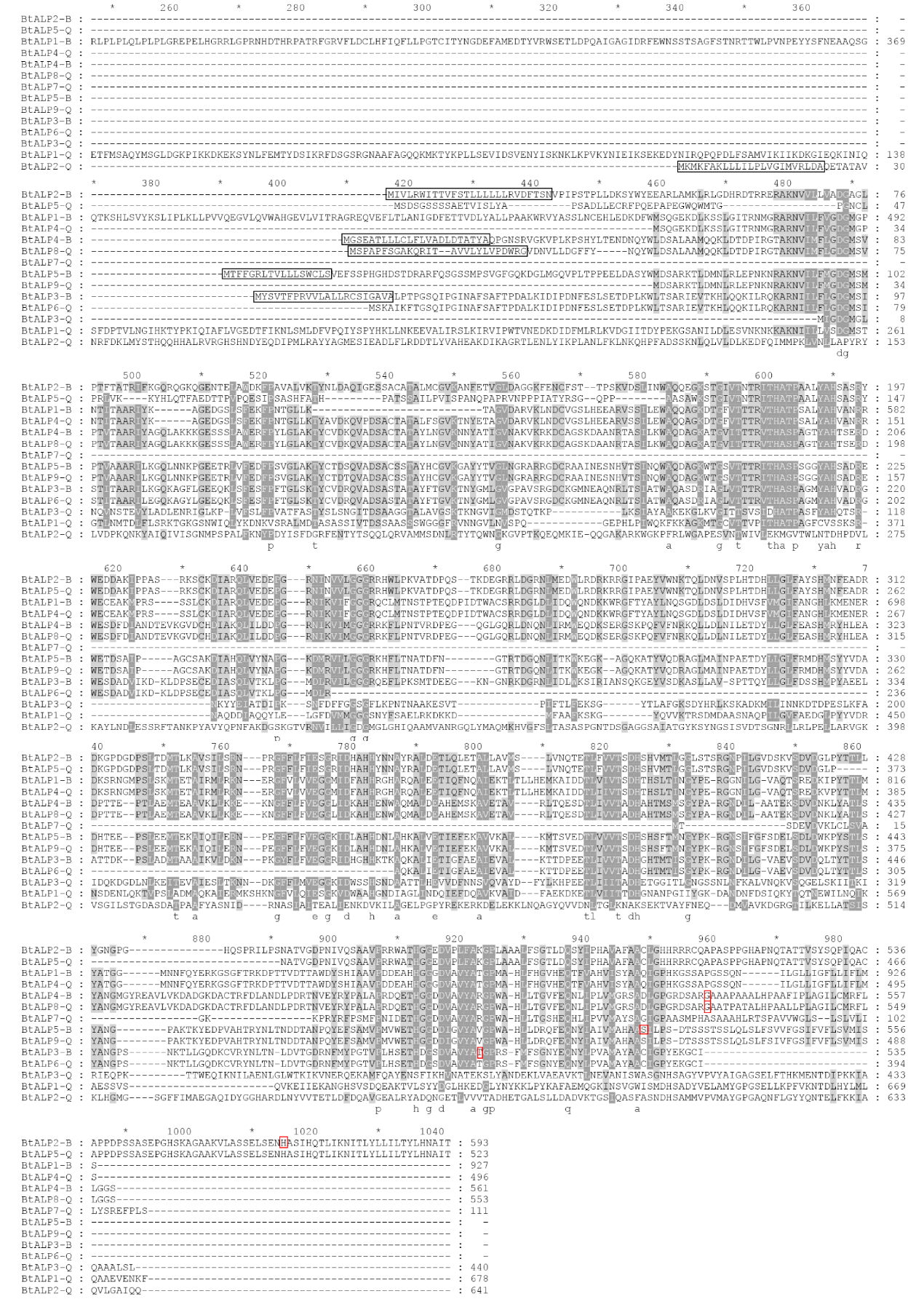


Fig.S.2 Multiple sequence alignments of ALP proteins from *B. tabaci* MEAM1 and MED based on CLUSTALW. Black boxes indicate the signal peptide in periplasmic ALP proteins. Red boxes indicate the GPI-anchoring site in periplasmic ALP proteins.
